# Protein NS4A of ZIKV Inhibits Glycolytic Flux by Targeting Enolase-1

**DOI:** 10.3390/cimb48050469

**Published:** 2026-05-01

**Authors:** Rui Ma, Kexin Zhang, Zhengfei Qi, Lina Wang, Qingshan Bill Fu

**Affiliations:** 1 School of Chinese Materia Medica, Nanjing University of Chinese Medicine, Nanjing 210023, China; marui1058@zidd.ac.cn (R.M.); zhangkexin1057@zidd.ac.cn (K.Z.); wanglina1056@zidd.ac.cn (L.W.); 2 Shanghai Institute of Materia Medica, Chinese Academy of Sciences, Shanghai 201203, China; 3 Zhongshan Institute for Drug Discovery, Shanghai Institute of Materia Medica, Chinese Academy of Sciences, Zhongshan 528400, China; yc57577@um.edu.mo; 4 State Key Laboratory of Quality Research in Chinese Medicine, Institute of Chinese Medical Sciences, University of Macau, Taipa, Macao 999078, China

**Keywords:** ZIKV virus, NS4A, ENO1, enzymatic activity, glycolysis

## Abstract

NS4A plays a role in forming the flavivirus replication complex, which inhibits apoptosis in host cells by inducing autophagy, thereby promoting viral replication. The host protein ENO1 interacts with NS4A, but the precise mechanism underlying this interaction and its role in viral replication remain unclear. In this study, we identified ZIKV NS4A^1–73^ as a key regulator of replication and infection cycles in both temporal and spatial dimensions. Through surface plasmon resonance (SPR) analysis, we demonstrated that ENO1 directly interacts with NS4A^1–73^. This critical binding inhibits the enzymatic activity of ENO1 and reduces cellular lactate and ATP production. Our findings suggest that ZIKV NS4A may effectively impede cellular metabolism by targeting the host factor ENO1, thus disrupting the glycolytic process. This insight could open new avenues for targeting ZIKV and similar viruses in therapeutic strategies.

## 1. Introduction

Zika virus (ZIKV) is a significant arbovirus that belongs to the same family as yellow fever virus (YFV), West Nile virus (WNV), Japanese encephalitis virus (JEV), and dengue virus (DENV) [1]. ZIKV is mainly transmitted through mosquito bites, but it has also been reported to be transmitted from mother to child and via sexual contact between humans [2,3,4]. Clinical manifestations of patients with ZIKV infection include fever, rash, conjunctivitis, arthritis, and muscle pain [5]. In addition, ZIKV can cause severe neurological complications, including placental insufficiency, fetal central nervous system damage, microcephaly, and even fetal death [6]. Currently, a specific treatment or vaccine for ZIKV infection has not been developed, and effective antiviral therapy is currently unavailable [7]. Therefore, it is essential to identify the host factors involved in ZIKV infection and to elucidate the molecular mechanisms underlying its pathogenesis, which will promote the creation of antiviral therapies and vaccines that are effective and safe.

Flaviviruses synthesize a long polyprotein, which a protease divides into three structural proteins and seven non-structural proteins. The capsid protein (C), precursor membrane protein (prM), and envelope protein (Env) are the three structural proteins. The proteins NS1, NS2A, NS2B, NS3, NS4A, NS4B, and NS5 are the seven non-structural ones [8]. The structure and function of most ZIKV proteins remain poorly characterized, but they are expected to be comparable to those of other flaviviruses such as DENV [9,10,11]. Structural proteins are key in the formation of viral particles and the process of viruses entering cells. Nonstructural proteins control the replication of the virus’s genome and influence the host’s immune reactions [12].

ZIKV NS4A consists of 127 residues, and like DENV NS4A, the topology and secondary structure of the mature protein contain a water-soluble N-terminal cytoplasmic domain (residues 1–48) and three predicted transmembrane (pTM) fragments [13,14]. pTM1 and pTM3 are the only ones that penetrate the membrane, with N-telopeptide (1–48) being related to cytopathic activity [15,16,17]. The multifaceted role of NS4A in the process of host cell infection has been reported. NS4A was prominently recognized as the primary inhibitor of RLR-mediated type I interferon production by interfering with the interaction between MAVS and MDA5 or RIG-I [18]. In human neural stem cells (NSCs), NS4A can enhance mitochondrial fission, which is associated with neuronal death induced by the ZIKV virus. Furthermore, NS4A and NS4B act together to cause damage. They disrupt the Akt-mTOR signaling pathway, affecting neuronal development, inducing autophagy, and promoting increased viral replication [19,20]. In addition, scientists also found that NS4A will directly interact with ANKLE2 protein, which is related to microcephaly in humans. After NS4A causes issues, the division of neural stem cells and the development of the brain will be disrupted [21]. NS4A is capable of moving to mitochondria, triggering mitochondrial fission and mitochondrial autophagy, and inhibiting mitochondria-associated antiviral protein (MAVS)-mediated interferon I (IFN) [22]. These results indicate that ZIKV NS4A plays a role in evading host immunity, viral pathogenesis, and the regulation of mitochondrial morphological and functional aspects, but the specific mechanism of NS4A virus replication is still unclear.

Enolase-1 (ENO1) is a multifunctional protein that acts not only as an α-phosphoenolase but also as an important glycolytic enzyme in eukaryotic and prokaryotic cells [23]. In addition, ENO1 functions as a neuronal survival factor and as a heat-shock protein HSP48 [24,25]. Moreover, ENO1 serves as a molecular marker in the diagnosis of various cancers [26,27,28,29]. Through alternative translation, messenger RNA (mRNA) produced by the ENO1 gene can be decoded to form a 37 kD protein called MBP-1. This protein stays in the nucleus, and it will attach to the P2 promoter region of the c-myc gene, thus inhibiting the transcription of proto-oncogenes. And it can also be translated into the 47 kD protein ENO1, which is localized to the cytosol to participate in glycolysis and other physiological and pathological processes [30]. The host protein ENO1 interacts with dengue virus NS4A [31,32]. This interaction is of great significance for further elucidating the regulatory mechanism of NS4A during flavivirus replication. However, whether this interaction also exists in ZIKV remains to be studied. Moreover, the molecular details and regulatory consequences of the potential interaction between ZIKV NS4A and ENO1 require further investigation.

The presence of the tail’s N-terminal and pTM1 domain of NS4A leads to significant overlap in the regions involved in homooligomerization and heterooligomerization. NS4A is combined by itself or mixed with other things to form a complex, which is extremely important for the virus to successfully replicate itself and arrange the time and location of each step in the whole infection process, and this represents a potential target for antiviral drug discovery [33,34,35,36]. Therefore, in this study, we chose to express a purified ZIKV NS4A (1–73) fragment and examined its in vitro interaction with ENO1 using surface plasmon resonance (SPR). In addition, the effect of NS4A^1–73^ binding on ENO1 enzymatic activity was assessed. Finally, the effect of NS4A^1–73^ on glycolytic cell flux was determined by measuring lactate production and ATP content. The results demonstrated that NS4A^1–73^ directly binds to ENO1, significantly inhibits its enzymatic activity, reduces lactate and ATP production, and suppresses cellular glycolytic flux.

## 2. Materials and Methods

### 2.1. Construction of Recombinant Plasmids

pET-21b(+) vector was used to produce NS4A^1–73^ protein, which is designed with a His tag, and NdeI/XhoI was used as the restriction enzyme for cutting. The ENO1 was ligated into the pQE-60 vector with a His tag. BamHI/BglII was the digestion site, and these two enzymes generate compatible cohesive ends but are not isoschizomers. In this particular cloning strategy, ligating the compatible ends results in the elimination of both restriction sites, thereby preventing subsequent recleavage. Furthermore, the pcDNA3.1(+) vector was utilized to produce NS4A^1–73^ with a FLAG tag and ENO1 with an HA tag, using BamHI and XhoI as the digestion sites for this process. All recombinant plasmids were synthesized by Genscript (Nanjing, China).

### 2.2. Expression and Purification of NS4A^1–73^

A recombinant plasmid designated NS4A^1–73^ was introduced into the *E. coli* strain BL21 (DE3), and permitted it to grow and express the protein in LB medium. Then, Induction was performed with 1 mM IPTG at 37 °C overnight to produce the protein. After induction, the cells were collected and resuspended in lysis buffer A (20 mM Tris-HCl, 300 mM NaCl, 15 mM sodium cholate, pH 8). They were then subjected to disruption using ultrasonic waves. The lysed cell fluid is subjected to centrifugation at a speed of 12,000× *g* for 40 min, after which the supernatant is collected. The protein supernatant was incubated with a nickel-nitrilotriacetic acid (Ni-NTA) chromatography column at 4 °C, and a gradient concentration imidazole elution strategy was adopted: first, an imidazole eluent with a low concentration (buffer A containing imidazole) was used to remove the heteroprotein, and then an imidazole eluent with a high-concentration was used to achieve specific elution of the target protein [37]. The experiment involves eluting the protein using 1–2 column volumes of elution buffer, with the eluates containing 25 mM, 50 mM, 100 mM, 200 mM, 300 mM, and 500 mM imidazole, respectively. Protein was further purified by size exclusion chromatography, with elution buffer containing 1 mM DTT and HBS-EP+ buffer, pH 7.4.

### 2.3. Expression and Purification of ENO1

The method of isolating the ENO1 protein is the same as that used to isolate NS4A^1–73^ before. The difference was that lysing cells with buffer B (50 mM NaH_2_PO_4_, 300 mM NaCl, pH 8.0) yielded better results. The cell lysate was centrifuged at 12,000× *g* for 40 min, and the supernatant was collected. The supernatant from cell lysis was bound to a Ni-NTA chromatography column. Low and high concentrations of imidazole were used to elute the heteroprotein and the protein of interest, respectively, to achieve separation. The experiment involves eluting the protein using 1–2 column volumes of elution buffer, with the eluates containing 25 mM, 50 mM, 100 mM, 200 mM, 300 mM, and 500 mM imidazole, respectively. This protein was purified by size exclusion chromatography, using an eluent containing 1 mM DTT and HBS-EP+ buffer, pH 7.4.

### 2.4. Surface Plasmon Resonance (SPR) Assay

Surface plasmon resonance (SPR) analysis was carried out using a Biacore T200 instrument (GE Healthcare, Chicago, IL, USA). ENO1 protein (120 μg/mL) was mixed with GTP-γ-S, diluted to 50 μg/mL with sodium acetate buffer with pH 4.0, and then immobilized on a CM5 sensor chip as ligand. NS4A^1–73^ was diluted to 1, 2, 4, 8, and 12 μM with buffer (1 mM DTT, HBS-EP+ buffer, pH 7.4). The dynamic characteristics were measured using a multi-cycle dynamic program. The binding time is set to 180 s, and the dissociation time is set to 300 s. In the experiment, the surface regeneration was carried out by using glycine hydrochloride buffer with a pH of 1.5, and each test was run for at least five cycles. The data from the sensor diagram were processed using Biacore T200 evaluation software version 3.0. The binding rate constant (ka), dissociation rate constant (kd), and equilibrium dissociation constant (KD) were calculated by fitting the curve with a 1:1 binding model [38].

### 2.5. ENO1 Activity Assay

A direct spectral method was employed to measure the activity of ENO1. The increase in absorbance at 240 nm was recorded, indicating the conversion of phosphoenolpyruvate (PEP). The experiment was conducted according to a previously published procedure, with minor modifications [39]. The study sets up a control group and an experimental group. In the control group, the reaction system buffer was thoroughly mixed with ENO1 (final concentration 120 nM) and 2-phosphoglycerate (final concentration 1 mM), with a total reaction volume of 100 μL. In the experimental group, the reaction system buffer was thoroughly mixed with ENO1 (final concentration 120 nM), NS4A^1–73^ protein (final concentration 8 μM), and 2-phosphoglycerate (final concentration 1 mM), with a total reaction volume of 100 μL. The change in OD_240_ absorbance from 0 to 40 min at room temperature was measured to observe ENO1 enzyme activity. After determining that the NS4A^1–73^ protein inhibited enzyme activity, to demonstrate that the inhibition of ENO1 enzyme activity by the NS4A^1–73^ protein was significant, sodium fluoride (NaF), an enolase inhibitor, was used as a control [40]. Nonlinear regression was conducted using a four-parameter model to fit the inhibition curves. The software GraphPad Prism is used in the analysis.

### 2.6. Culturing and Transfection of 293F Cells

Cryopreserved 293F cells were resuscitated in a 37 °C water bath, and after removing DMSO, they were resuspended in preheated 293 expression medium and suspension culture at 37 °C. The first passage was performed after 96 h of culture, and then every 48 h thereafter until cell viability was steadily restored, marked by doubling of cell density within 24 h. When the cells grew to a density of 1.6 × 10^6^/mL and were in good shape. A transfection reagent known as polyethylenimine (PEI) was used to treat the cells. The method is to use 1 microgram of plasmid for every 1.6 × 10^6^ cells. In this process, PEI and plasmid are mixed together according to the weight ratio of 3:1 [41]. These cells were divided into four groups: the first group was a blank control and was transfected with 20 μg ddH_2_O; the second group was transfected with 20 μg NS4A^1–73^-FLAG-pcDNA3.1(+); the third group was transfected with 20 μg ENO1-HA-pcDNA3.1(+); the fourth group was cotransfected with 10 μg NS4A^1–73^-FLAG-pcDNA3.1(+) and 10 μg ENO1-HA-pcDNA3.1(+). Western blot showed that both NS4A^1–73^ and ENO1 proteins were successfully expressed 36 h after transfection.

### 2.7. Detection of Lactate Production and ATP Content

The Lactate Assay kit supplied by Solarbio (Beijing, China) was utilized to quantify the lactate levels in the culture medium. The ATP level in cells was also measured by the Luciferase-dependent ATP Assay Kit of Beyotime (Shanghai, China). The measured lactate production and ATP content were adjusted according to the number of cells.

### 2.8. Statistical Analysis

GraphPad Prism 9.1.0 was used for statistical analysis, and the data are presented as mean ± SD. One-way ANOVA was used when comparing multiple sets of data. *p* < 0.05 was considered to be significant.

### 2.9. Western Blot

Protein lysates were prepared using RIPA buffer (Beyotime) supplemented with a protease inhibitor cocktail. Equal amounts of protein (16 μg per lane) were separated by 12.5% SDS-PAGE. The protein in the gel was then transferred to a PVDF membrane. The transfer process lasted for 2 h at 90 V. After transfer, the PVDF membrane was washed with TBST buffer three times for 5 min each. Subsequently, membranes were blocked with 5% non-fat milk in TBST for 2 h at room temperature and then incubated with primary antibodies overnight at 4 °C. The following primary antibodies were used: FLAG tag Monoclonal antibody (catalog number: 66008-4-Ig, Proteintech, Rosemont, IL, USA, 1:5000), HA tag Monoclonal antibody (catalog number: 66006-2-Ig, Proteintech, 1:10,000), and GAPDH Mouse Monoclonal Antibody (catalog number: AF0006, Beyotime, 1:1000). After thoroughly washing off the unbound primary antibody with TBST, the membrane was incubated with HRP-conjugated secondary antibody (anti-mouse IgG, catalog number: A0216, Beyotime, 1:1000) for 2 h at room temperature. Protein bands were visualized using the Fg Super Sensitive ECL Luminescence Reagent kit (Meilunbio, Dalian, China) and detected with the Chemiluminescence Imaging System (Tanon, Shanghai, China).

## 3. Results

### 3.1. Purification of NS4A^1–73^

**Figure 1:**
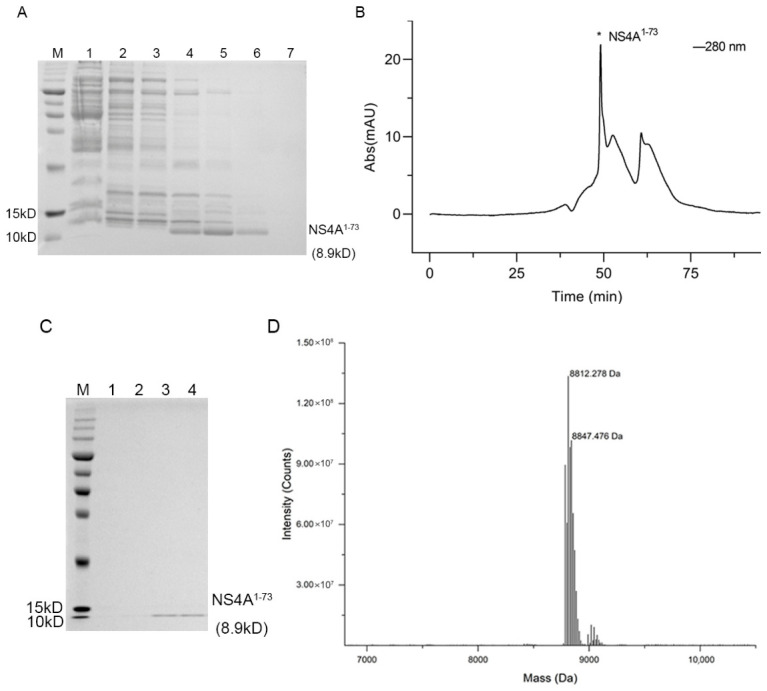
Purification of NS4A^1–73^. (**A**) Results of NS4A^1–73^ elution on 12.5% SDS-PAGE by different concentrations of imidazole buffer. M: Prestained protein ladder, 1: Flow through of Ni-NTA chromatography column, 2–7: Elution with 25, 50, 100, 200, 300, and 500 mM imidazole, respectively. (**B**) Size exclusion chromatogram of NS4A^1–73^. *: The elution peak position of protein NS4A^1–73^. (**C**) The figure shows the results of NS4A^1–73^ eluted peaks on 12.5% SDS-PAGE. M: Prestained protein ladder, 1–4: Purified NS4A^1–73^, around 8.9 kD. (**D**) Result of the mass spectrometry for the NS4A^1–73^ protein.

The NS4A^1–73^ with an N-terminal 6× His tag was constructed in the pET-21b(+) plasmid vector, and the molecular weight of the protein is approximately 8.9 kD. The NS4A^1–73^ protein was expressed in *E. coli* and eluted using imidazole. SDS-PAGE results of the imidazole gradient elution showed a distinct protein band corresponding to NS4A^1–73^ around 10 kD, and NS4A^1–73^ was successfully eluted from the column by using a 100 mM imidazole solution. Some co-eluted contaminant proteins were also present in the same fractions (Figure 1A). The 200 mM imidazole fractions containing NS4A^1–73^ were further purified by size-exclusion chromatography to obtain a more homogeneous protein sample. The first major peak on the chromatogram corresponds to NS4A^1–73^ (Figure 1B). The purified NS4A^1–73^protein was confirmed by SDS–PAGE analysis (Figure 1C). To further confirm, a portion of the sample from the first peak was analyzed using mass spectrometry to identify the protein’s molecular weight. The results showed that the observed molecular weight corresponded to the target protein NS4A^1–73^ with a molecular weight of 8.9 kD, confirming it as NS4A^1–73^ (Figure 1D). Uncropped, full-length images of the gels shown in this figure are available in Figure S1.

### 3.2. Purification of ENO1

**Figure 2:**
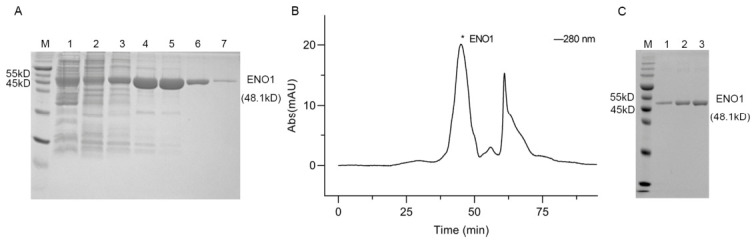
Purification of ENO1. (**A**) Results of ENO1 elution on 12.5% SDS-PAGE by different concentrations of imidazole buffer. M: Prestained protein ladder, 1: Flow through of Ni-NTA chromatography column, 2–7: Elution with 25, 50, 100, 200, 300, and 500 mM imidazole, respectively. (**B**) Size exclusion chromatogram of ENO1. *: The elution peak position of protein ENO1. (**C**) The figure shows the results of ENO1 eluted peaks on 12.5% SDS-PAGE. M: Prestained protein ladder, 1–3: Purified ENO1, around 48.1 kD.

ENO1 with an N-terminal 6 × His tag was constructed in the pQE-60 plasmid vector, and the molecular weight of the protein is approximately 48.1 kD. Following induced expression in *E. coli*, the ENO1 protein was purified separately on a Ni^2+^-NTA affinity column. In order to separate ENO1 from host-derived contaminants, we used a method: let the protein be washed out step by step in the buffer containing different concentrations of imidazole. SDS–PAGE analysis showed that a distinct ENO1 protein band appeared between 45 kD and 55 kD, and ENO1 was predominantly eluted at 100 mM imidazole (Figure 2A). ENO1 was further purified by size exclusion chromatography, and a high-purity product was obtained. In the obtained chromatogram, ENO1 was detected in the first elution peak (Figure 2B), and its expression and purity were confirmed by SDS–PAGE analysis (Figure 2C). Uncropped, full-length images of the gels shown in this figure are available in Figure S2.

### 3.3. NS4A^1–73^ Has a Specific Binding Activity to ENO1

**Figure 3:**
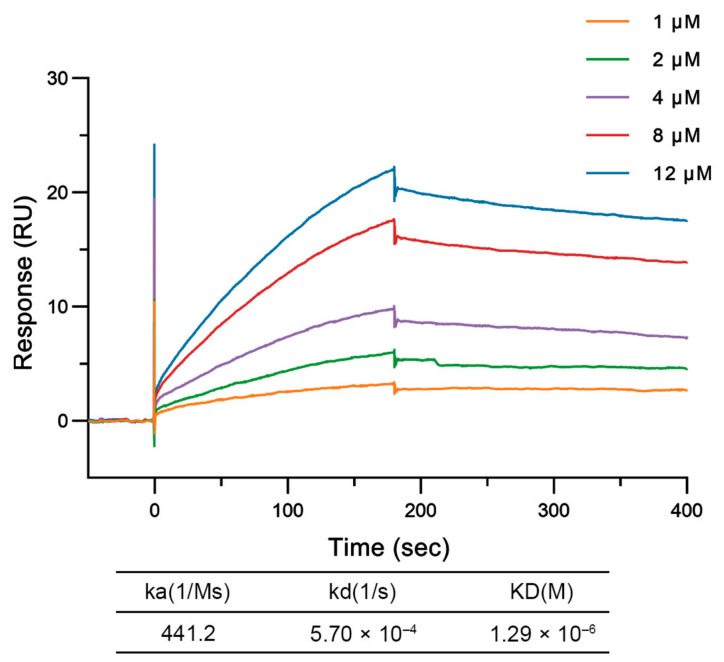
The binding and dissociation sensorgrams of NS4A^1–73^ and ENO1. ENO1 was immobilized on the chip as a ligand. NS4A^1–73^ protein was used as a flowing analyte. The running buffer was an HBS-EP+ buffer with 1 mM DTT during the multicycle kinetic analysis process.

In order to study the interaction between NS4A^1–73^ and ENO1, we first fixed ENO1 on a Biacore CM5 sensor chip. Then, we put different concentrations of NS4A^1–73^ into the flow system to see if they are tightly combined. NS4A^1–73^ and ENO1 were diluted in HBS-EP+ buffer with 1 mM DTT. A transient signal spike at the beginning of the injection (0 s) was attributed to the presence of DTT in the buffer. As a reducing agent containing two sulfhydryl groups, DTT can transiently increase the response signal when the sample contacts the chip surface, causing a short-lived peak in the response curve. However, this artifact did not affect the final binding kinetics. After we subtract the blank control of 0 μM protein concentration, we analyze the sensor data and use a model called the 1:1 binding model to fit it. This analysis helps us get the association constant (ka) and dissociation constant (kd), with KD = kd/ka. The results show that there is a strong interaction between NS4A^1–73^ and ENO1, with a KD of 1.29 × 10^−6^ M (Figure 3).

### 3.4. NS4A^1–73^ Can Inhibit ENO1 Activity

**Figure 4:**
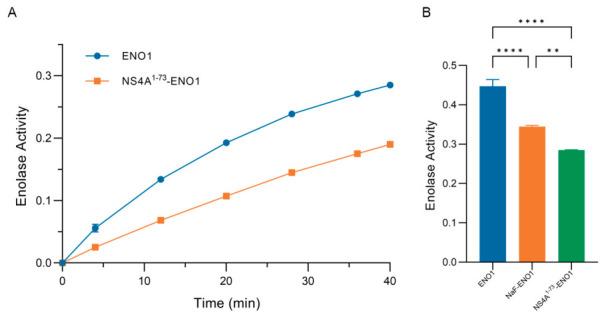
Enzyme activity test results. (**A**) Inhibitory effect of NS4A^1–73^ on ENO1 over time. (**B**) The degree of inhibition of ENO1 by NS4A^1–73^ was compared with that of NaF (** *p* < 0.01 and **** *p* < 0.0001). The concentration of NS4A^1–73^ is 8 μM, ENO1 is 120 nM, and NaF is 5 mM. This experiment was done three times; the results are expressed as mean and standard deviation.

To determine if the combination of NS4A^1–73^ and ENO1 will affect the functions of ENO1, the purified ENO1 was assayed in the presence and absence of NS4A^1–73^. Over time, NS4A^1–73^ gradually inhibited the activity of ENO1 (Figure 4A). When enzyme activity was measured at 30 min, NS4A^1–73^ inhibited ENO1 more strongly than the known inhibitor NaF (Figure 4B) (** *p* < 0.01 and **** *p* < 0.0001).

### 3.5. NS4A^1–73^ Inhibits Cell Glycolysis

**Figure 5:**
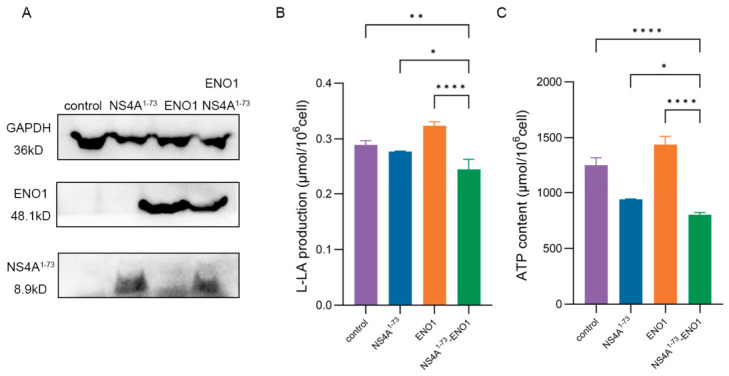
Lactate production and ATP content were detected and analyzed in cells. (**A**) Western blot results for NS4A^1–73^ and ENO1 in 293F cells. (**B**) Lactate production was detected and analyzed. (**C**) The luminous intensity was measured by the ATPLite detection method to calculate the ATP content. (* *p* < 0.05, ** *p* < 0.01, and **** *p* < 0.0001). Lactate and ATP levels were both normalized by cell number.

Equal volumes of plasmids encoding NS4A^1–73^ and ENO1 were individually transfected into 293F cells, while a 1:1 mixture of NS4A^1–73^ and ENO1 plasmids was co-transfected into an equivalent number of cells. ddH_2_O served as a negative control. Western blot analysis showed that NS4A^1–73^ and ENO1 were successfully expressed in 293F cells (Figure 5A). Cellular lactate and ATP levels were measured to evaluate the effect of NS4A^1–73^ on the glycolytic process. Compared with the NS4A^1–73^ group, the lactate concentration was significantly higher in the ENO1-expressing group (Figure 5B). ATP levels measured by the ATPLite luminescent assay were also significantly higher in the ENO1 group than in the NS4A^1–73^ group (Figure 5C). In the co-expression group (NS4A^1–73^-ENO1), both lactate and ATP levels were significantly reduced compared with cells expressing ENO1 alone. A significant difference can also be observed by comparing the results of the co-transfection group and the control group. These findings indicate that NS4A^1–73^ reduces lactate and ATP production in cells. Collectively, these results demonstrate that NS4A^1–73^ inhibits cellular glycolytic flux. All differences were statistically significant. (* *p* < 0.05, ** *p* < 0.01, and **** *p* < 0.0001) Uncropped, full-length images of Western blots shown in this figure are available in Figure S3.

## 4. Discussion

The non-structural protein of ZIKV is an indispensable factor in the process of viral replication. However, the current research on ZIKV NS protein mainly focuses on NS1, NS2B, NS3, NS5, and other proteins, while NS4A has been relatively less studied. Disruption of the helical structure in the N-terminal region of NS4A significantly inhibits viral replication, suggesting that this domain may serve as a potential target for in vitro antiviral screening [37]. By comparing flavivirus–human networks, ZIKV NS4A was found to interact with many host proteins involved in mitochondrial function, intracellular transport, oxidative respiration, autophagy, and immune regulation [42]. To investigate the molecular mechanism of viral replication regulation, NS4A (1–73) was selected as the starting point for this study. It will be of great significance to elucidate the role of NS4A in viral replication and to find new antiviral targets.

ENO1, as a multifunctional glycolytic enzyme, plays an important role in various cellular processes. In addition to functioning as a glycolytic enzyme to regulate the transcription of the Sendai virus genome [43], ENO1 can interact with viral proteins during Dengue virus (DENV) infection to modulate replication [32]. We propose that ZIKV NS4A^1–73^, as an ER membrane-associated protein, can similarly bind to ENO1 and influence glycolytic flux. This hypothesis has now been clearly stated and will guide future experiments.

In DENV, the interaction between NS4A and ENO1 has only been confirmed using protein immunoprecipitation techniques, and the mechanism and effects of this interaction have not yet been investigated. But in this study, we propose that NS4A^1–73^ can affect the glycolysis process by targeting ENO1. Through the study of the NS4A^1–73^ protein, it was found that NS4A^1–73^, as an important non-structural protein of ZIKV, directly interacts with the host factor ENO1. This interaction has been confirmed through assays to inhibit the enzymatic activity of ENO1 and can suppress glycolytic flux in cells by targeting ENO1, thereby affecting cellular metabolism. These studies provide a new avenue for further investigation into the role of NS4A during ZIKV infection of cells.

Protein–protein interactions are fundamental to cellular physiology. NS4A likely facilitates viral infection by interacting with host factors, some of which may also act in defense by counteracting NS4A function. In this study, the molecular mechanism and specific effects of ZIKV NS4A in viral replication were elucidated. These findings provide new insights into the molecular mechanisms of ZIKV NS4A in viral replication and pathogenesis, offering potential targets for antiviral drug development and vaccine design for the prevention or treatment of ZIKV infection.

This study still has some limitations. The protein structure of NS4A remains unclear, and the interaction targets need to be further confirmed through mutations. The interaction between NS4A^1–73^ and ENO1 was demonstrated in vitro and in overexpression systems, rather than in the context of actual viral infection. These considerations do not invalidate the conclusion that NS4A^1–73^ interacts with ENO1, but they do emphasize the necessity of infection-based studies. Given the critical dependence of developing neural tissues on glycolytic metabolism, it is conceivable that NS4A-mediated inhibition of ENO1 may contribute to metabolic dysregulation during fetal brain development, although this hypothesis requires validation in neural and in vivo models. Future structure–function studies using NS4A mutants will be critical to define the molecular determinants of ENO1 inhibition and to evaluate how these interactions may contribute to the neurological outcomes associated with Zika virus infection.

## Data Availability

The original contributions presented in this study are included in the article/Supplementary Material. Further inquiries can be directed to the corresponding author.

## Supplementary Materials

The following supporting information can be downloaded at: https://www.mdpi.com/article/10.3390/cimb48050469/s1.
