# Supplementary material for: Protein NS4A of ZIKV Inhibits Glycolytic Flux by Targeting Enolase-1
Source: Curr Issues Mol Biol. 2026 May 1;48(5):469. doi: 10.3390/cimb48050469 (PMC13204584; doi:10.3390/cimb48050469)
Supplement: Supplementary file 1 [file cimb-48-00469-s001.zip › cimb-4227915-supplementary.pdf]

Supplementary figures

Figure S1 A B

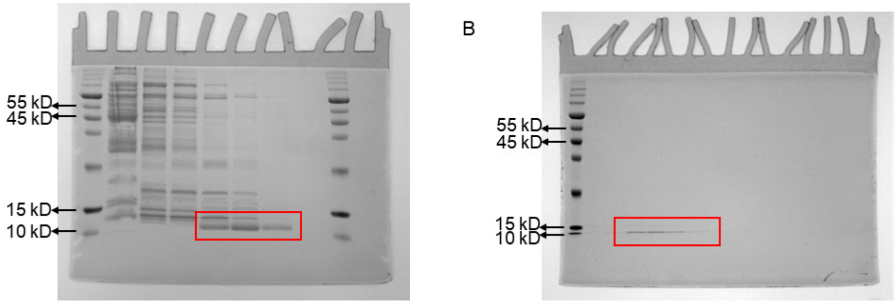

Figure S1. The original images of NS4A$^{1-73}$ gel electrophoresis. (A) Original gel electrophoresis image of NS4A$^{1-73}$ eluted with imidazole gradient. (B) Original gel electrophoresis image of SCE-purified NS4A$^{1-73}$.

Figure S2 A B

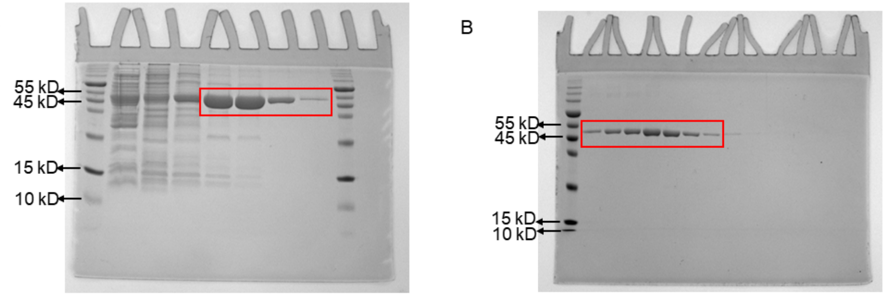

Figure S2. The original images of ENO1 gel electrophoresis. (A) Original gel electrophoresis image of ENO1 eluted with imidazole gradient. (B) Original gel electrophoresis image of SCE-purified ENO1.

Figure S2 A B C D E F G H I

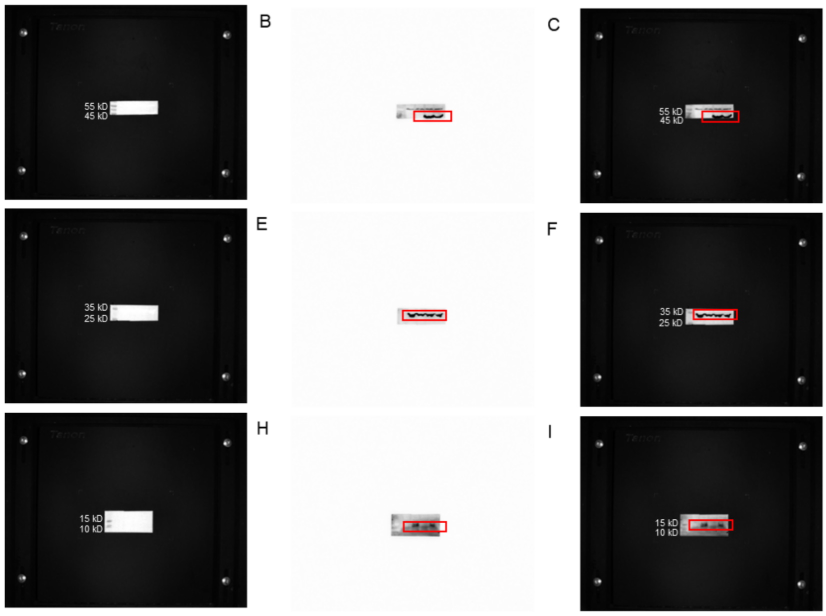

Figure S3. The original images of the Western blots. (A) Original bright-field image of

ENO1. (B) Original white light image of ENO1. (C) Original merged image of ENO1. (D) Original bright-field image of GAPDH. (E) Original white light image of GAPDH. (F) Original merged image of GAPDH. (G) Original bright-field image of NS4A$^{1-73}$. (H) Original white light image of NS4A$^{1-73}$. (I) Original merged image of NS4A$^{1-73}$.
